# Capturing in-field root system dynamics with RootTracker

**DOI:** 10.1093/plphys/kiab352

**Published:** 2021-07-27

**Authors:** Jeffrey J Aguilar, Matt Moore, Logan Johnson, Rachel F Greenhut, Eric Rogers, Drew Walker, Fletcher O’Neil, Jake L Edwards, Jake Thystrup, Sam Farrow, Jesse B Windle, Philip N Benfey

**Affiliations:** 1 Hi Fidelity Genetics, Durham, NC USA; 2 Duke University, Department of Biology, Durham, NC, USA and Howard Hughes Medical Institute (HHMI)

## Abstract

Optimizing root system architecture offers a promising approach to developing stress tolerant cultivars in the face of climate change, as root systems are critical for water and nutrient uptake as well as mechanical stability. However, breeding for optimal root system architecture has been hindered by the difficulty in measuring root growth in the field. Here, we describe the RootTracker, a technology that employs impedance touch sensors to monitor in-field root growth over time. Configured in a cylindrical, window shutter-like fashion around a planted seed, 264 electrodes are individually charged multiple times over the course of an experiment. Signature changes in the measured capacitance and resistance readings indicate when a root has touched or grown close to an electrode. Using the RootTracker, we have measured root system dynamics of commercial maize (*Zea mays*) hybrids growing in both typical Midwest field conditions and under different irrigation regimes. We observed rapid responses of root growth to water deficits and found evidence for a “priming response” in which an early water deficit causes more and deeper roots to grow at later time periods. Genotypic variation among hybrid maize lines in their root growth in response to drought indicated a potential to breed for root systems adapted for different environments. Thus, the RootTracker is able to capture changes in root growth over time in response to environmental perturbations.

## Teaser

RootTracker, a technology that employs impedance touch sensors, enables monitoring in-field root growth over time.

## Introduction

Yield stability in agriculture is a major challenge in the face of climate change, necessitating crop varieties that are resilient to stressful environmental conditions such as drought. Optimizing root system architecture offers a promising approach to developing stress tolerant cultivars, as root systems are critical for water and nutrient uptake as well as mechanical stability. However, breeding for optimal root system architecture has been hindered by the difficulty in measuring root growth in the field. Current methods are laborious and not easily scaled. To enable the much broader use of root system architecture information in agronomic research, we have developed a device that overcomes these challenges.

Optimization of root system architecture could provide benefits beyond stress tolerance—it could even reduce greenhouse gas levels. Roots contribute to carbon sequestration in soil through exudates and cell wall material (Paul and Clark, 1996; Dakora and Phillips, 2002). An analysis of soil organic carbon in croplands indicates that modulating root growth can significantly impact greenhouse gas emissions mitigation (Paustian et al. 2016). Larger and deeper root systems can result in greater deposits of organic carbon compounds with longer mean residence times (Kell, 2012). Further, there are strong arguments that deeper root systems provide enhanced water and nitrogen uptake in many circumstances (Lynch, 2013). Thus, it is possible to optimize root systems for abiotic stress tolerance, nutrient uptake efficiency, and carbon sequestration, simultaneously.

To breed for optimal root systems, one needs to measure root growth in the field. Common methods used for root phenotyping in the field are shovelomics (Trachsel et al., 2011) and coring (Wasson et al., 2014). While advances in image analysis (Das et al., 2015; Colombi et al., 2015) have allowed for higher throughput of shovelomics phenotyping, both shovelomics and coring are destructive and allow for only a single snapshot of root growth of an individual plant. Another option, minirhizotrons—clear tubes inserted and left in the soil with imaging equipment periodically inserted—typically only image a small and localized subset of the root system (Rytter and Rytter, 2012). Among noninvasive methods (reviewed in Wasson et al. 2020), ground penetrating radar (Delgado et al., 2017) has been used to measure bulk root properties. However, to date, it has been limited to use with roots of relatively large diameter. Another approach involves charging a circuit with one electrode connected to the plant’s stem and the other to the soil. This technique has correlated capacitance with tree root length (Ellis et al., 2013) and root mass (Dalton, 1995; van Beem et al., 1998) of herbaceous crops, but did not provide a measure of growth rates.

One of the biggest advantages of the technology described herein compared to other methods is the ability to monitor root growth on a continuous basis in the field, enabling not just the measurement of root system architecture, but of root system dynamics—how root growth changes over time and responds to changes in the environment. Measuring root systems in 4D (space and time) has been achieved primarily in lab settings with techniques such as X-ray computed tomography, magnetic resonance imaging, and positron emission tomography (Atkinson et al., 2019). However, insights from the lab often do not translate to the field due to artificial laboratory conditions, and hence for the purposes of plant breeding or agronomic research, it is preferable to conduct field trials. This underexplored area offers promising opportunities for finding ways to improve stress tolerance (Arsova et al., 2020).

Although current methods for phenotyping roots have limitations, breeding for root traits has been shown to enhance plant performance (Tracy et al., 2019). In rice (*Oryza sativa* L.), steeper (Uga et al., 2011) and deeper (Hurd, 1974; Wasson et al., 2014) root systems were selected for water capture in deeper soil strata, and thicker primary root systems were selected for greater biomass in drought-prone areas. Identifying and connecting such phenotypic responses in field conditions to crop performance metrics such as yield stability (Wang et al., 2014) will aid in developing crop varieties robust to climate change.

Here, we describe the RootTracker, a technology that employs impedance touch sensors to monitor in-field root growth over time. We have used the RootTracker platform to measure root system dynamics of commercial maize (*Zea mays*) hybrids growing in both typical Midwest field conditions and under different irrigation regimes in water-controlled environments. In these experiments, we discovered remarkably rapid responses of root growth to water deficits. We found evidence for a “priming response” in which an early water deficit causes more and deeper roots to grow at later time periods. There was genotypic variation among hybrid maize lines in their root growth in response to drought, indicating a potential to breed for root systems adapted for different environments.

## Materials and methods

### RootTracker field installation

All fields were tilled prior to experiment installation. Raised beds were formed for trials at Massai Agricultural Services in Rancagua, Chile and at the Kearney Agricultural Research and Extension (KARE) Center located in Parlier, California. Similarly, furrows between rows were created prior to trial installation at Real Farm Research in Aurora, Nebraska. Standard fertilizer, weed and pest control for maize (*Z. mays*) were used based on the recommendation of each cooperator.

Within each row, RootTrackers were installed every two feet (every other plant, with the exception of Trial 2, where all plants spaced one foot apart within a row had a RootTracker). After RootTrackers were installed, seeds were sown by hand. In some trials, two seeds were sown and thinned to a single plant after emergence. In all trials, plants were spaced one foot apart within rows and between row spacing was 30 inches. See Supplemental Figures S5–S8 for field layout maps and Supplemental Table S1 for a summary of RootTracker trials.

RootTracker installation is easiest in softer, tilled soil. Tool-free hand installation (i.e. pushing them into the ground by hand) is seldom possible. After soil preparation, RootTrackers were set upright in the field. To minimize paddle bending during installation, we fabricated two-mm thick plastic guide rings to sleeve the paddles and keep them vertical during installation. The RootTrackers were installed using a gas-powered fence post driver in conjunction with a custom-welded fence post hammer that mates with the RootTracker’s center hole and the paddle tops. The process involves two operators, one operating the fence post driver as it rests on the hammer and another holding the hammer upright for proper vertical installation. As a RootTracker is pushed into the ground, the guide ring presses up against the underside of the RootTracker surface. The time to install a single RootTracker with a fence post driver varies between 30 seconds and one minute, depending on the compaction of the soil. The overall per-RootTracker time to set up a field experiment—which includes soil preparation, moving RootTrackers to the field, installation, battery plug in, base station setup, planting, and mapping unique device IDs with field location and experimental condition—is about 8–10 minutes, though typically these activities happen over several days in a staged fashion. In these experiments, the full setup with a team of six to eight people usually took two to three days.

Weather data for all trials are listed in Supplemental Table S2 (Globalmet 2021, University of California 2021, Menne et al. 2012a, Menne et al. 2012b, Clemson 2021). The soil found at the site of Trials 1 and 2 was an alluvial soil, the analysis of which can be found in Supplemental Figure S9. The soils found at Trials 3, 4 and Sorghum (*Sorghum bicolor*) Trial were Hanford Sandy Loam, Crete Silt Loam, and Norfolk Loam Sand, respectively (Soil Survey Staff, 2021).

### Data logging and communication

Each RootTracker records raw voltage measurements every five minutes. A radio module on the RootTracker (the RFM69 HCW) communicates data upon measurement through one of several radio frequencies ranging from 902 to 924.5 MHz to solar powered base stations. Each base station receives signals from specific frequencies. Aside from distinct frequencies, the radio modules further filter radio traffic by signals transmitted on designated networks and nodes within that network. In each experiment, the RootTrackers were pre-programmed to communicate on a unique node with an array of networks/frequencies. To minimize radio traffic interference among RootTrackers, the base stations transmitted correction time delays to the RootTrackers that ensured transmissions of all RootTrackers within a network were evenly spaced within each five-minute time window. The base stations compiled and compressed the received data in 15-minute increments and communicated via a cell modem to remote servers on Amazon Web Services (AWS). Data from AWS were regularly downloaded, parsed, processed, and analyzed on Hi Fidelity Genetics servers located in Durham, NC.

### RootTracker tagging and data culling

Each RootTracker was a priori tagged with identifiers derived from its unique node/network/frequency assignment as well as a unique hardware serial number, which were used to record the field location of each RootTracker. RootTrackers with no plant due to poor germination or premature death from physical damage (e.g. extreme weather or pest damage) were identified and excluded from all statistical calculations. The trials conducted in Rancagua, Chile also included a parallel technology development test, whereby two versions of the RootTracker, which differed in their electronics configurations, were compared for detection accuracy. Version 2, which comprised 881 of the 1,223 RootTrackers in Trial 1 (and 721 of 1,154 RootTrackers in Trial 2), incorporated modified capacitance charging circuitry, and demonstrated superior ground truth correlation as compared to the older design (Version 1; Supplemental Figure S10). Consequently, all data presented in this article from Trials 1 and 2 (with the exception of the Version 1 ground truth figure, Supplemental Figure S10B) used only data from Version 2 devices. The remaining trials (Trials 3, 4, and the Sorghum ground truth trial) exclusively had Version 2 devices. Trials 3, 4, and Sorghum had 1,457, 1,482, and 409 of these devices, respectively (see Supplemental Table S1 for more trial details). In all trials, each RootTracker had one plant. See Supplemental Data SetsS1, S2, S3, and S4 for a list of RootTrackers in Trials 1, 2, 3, and 4, respectively. See Supplemental Table S3 for a list of the number of RootTracker replicates (N) for each treatment/genotype combination of each of the five trials. RootTrackers that were nonfunctional at the outset due to installation damage or any other reason, or had missing tags or duplicate IDs, were excluded from the list. See the “Materials and methods” Root Detection Calculations for an explanation of further culling post analysis.

### Drought trial irrigation methods

The trials in Rancagua, Chile (Trials 1 and 2) were irrigated using pressure-compensating drip tape laid on top of each raised bed and secured under the bottom side of the RootTracker’s white plastic enclosure. Drip tape in rows associated with the same treatment were connected to a main water line with valves placed to allow for treatment-specific irrigation regimes by manually opening or closing valves. Irrigation time and duration were based on soil water holding capacity, estimated plant water demand during different plant growth stages, and average daily temperature, following standard maize irrigation methods of the cooperator. No rainfall events occurred throughout the course of these trials.

The trial at KARE Center (Trial 3) was irrigated using drip irrigation. Drip lines were placed in the furrow bottoms between raised beds to avoid pooling on top of beds. The reasoning for this placement was that ponding of water in this sandy loam soil can result in a silty hard crust on the soil surface. Total weekly crop water demand was estimated using the previous week’s crop evapotranspiration (ETc). ETc was estimated using weather data from the California Irrigation Management Information System (CIMIS) station located in Parlier and estimated plant water demand for different maize plant growth stages. To assess relative uniformity of drip water application amounts, the average drip emitter water output was checked at intervals applying a system pressure of 10 psi. This was estimated by measuring the amount of water emitted over a set time period for 12–16 emitters (varied at different times), with the evaluated emitters distributed across the entire field. Given this average volumetric output, the average water application rate of the system was calculated to be 0.19 inches per hour. This rate was used in conjunction with the estimated ETc to determine the total irrigation time per week. For example, according to the CIMIS station data and crop demand, ETc for the week of August 12, 2019 to August 18, 2019 was estimated to be 1.99 inches. Thus, given a watering rate of 0.19 inches per hour, the recommended watering time for the following week was 10.5 hours. The total irrigation time for a given week was distributed across multiple days to allow the soil to dry sufficiently between irrigation events and aid our ability to walk in the field to collect above ground plant measurements and observations.

### Ground truth root imaging method

In January of 2019, all RootTrackers in Trial 1 were excavated by hand using shovels, keeping the roots intact within a one-foot diameter and 8-inch depth around the RootTracker. Plants were cut above the brace roots and roots were carefully removed from RootTrackers, keeping track of the associated RootTracker, field location, genotype, and treatment. Roots were gently washed in large bins of water with mild detergent. After washing, roots were laid out to air dry. Dry roots were photographed in a photo area constructed to capture images with consistent lighting, focus, and distance from the roots. The imaging setup also provided consistent contrast between the root system and backdrop, allowing for accurate identification of image pixels containing root matter (Figure 2A).

The goal of the shovelomics image analysis was to approximate the amount of root matter in close proximity to the region of the RootTracker where paddle electrodes were located. For each image, a threshold pixel intensity was used to identify and count all root pixels, S, located in the region of paddle electrodes. Known dimensions of the RootTracker, consistent placement of the root system in the image frame, and a known pixel scale were used to identify this region (red rectangle in Figure 2B).

Shovelomics root pixel calculations for each RootTracker were compared with R, the daily root detection rate, time-averaged across the entire trial period calculated for each RootTracker. Correlations were generated via grouping R and S by genotype and calculating respective median values $\tilde{R}$ and $\tilde{S}$.

The same analysis process was utilized to compare root imagery with RootTracker detections from a sorghum trial (Supplemental Figure S2). The trial was performed in collaboration with Clemson University at the Pee Dee Research and Education Center in Darlington, South Carolina. As with all other data presented in this article, this trial consisted of Version 2 RootTrackers. The field installation, data logging, communication, tagging, and culling procedures were identical as in other trials, though no irrigation was used, and the RootTracker and plant spacing were both one foot within a row. The trial consisted of 409 plants in RootTrackers that were excavated and the roots analyzed.

### Root detection calculations and statistics

RootTracker detections are determined from processed raw voltage signals. Using direct current charging, each electrode is charged while all other sensors are grounded, and voltage at that electrode is measured multiple times with different charge times. Assuming a simple parallel resistor–capacitor circuit between a charged electrode and surrounding grounded electrodes, an estimate of capacitance and resistance is calculated using measured voltages.

(1) $$\mathrm{Res}=\frac{{\mathrm{Res}}_{\mathrm{VD}}V_{2}}{V_{s}-V_{2}}$$

Res is the resistance; ${\mathrm{Res}}_{\mathrm{VD}}$ is the voltage divider resistance; $V_{2}$ is the measured voltage of the electrode for a time sufficiently long to assume no capacitance effect on voltage measurement, and $V_{s}$ is the source voltage.

(2) Cap= -TC(Res+ResVD)Res·ResVDln⁡(1-(V1/Vs)(Res+ ResVD)/Res)

Cap is capacitance, $T_{C}$ is the short time used to charge the electrode, $V_{1}$ is the measured voltage of the electrode when charged for $T_{C}$ time.

A sample raw voltage signal from an electrode charged for one microsecond on a RootTracker in Trial 2 in the water-limited treatment (Supplemental Figure S1A) illustrates a time period when the soil adjacent to the electrode was previously irrigated and was drying until another irrigation event on February 22, 2019. The daily fluctuations in voltage represent signal sensitivity to daily changes in soil temperature and moisture. The corresponding calculation of resistance and capacitance for the electrode during this time period (Supplemental Figure S1B) elucidates how the resistance increases and capacitance decreases during drying and vice versa during events of wetting along a characteristic curve in R-C space. We have identified signature fluctuations in the resistance/capacitance space that indicate root growth activity near the sensors once signal changes have been normalized across all electrodes. In particular, a root detection is often characterized by the ratio of change in resistance versus capacitance as being smaller compared to regular changes associated with wetting. We suspect that this is associated with roots being not as conductive as watered soil when they touch electrodes. We utilized both the direction of signal change in R-C space as well as the magnitude of the change relative all other electrodes of the RootTracker to account for global electrical changes in the soil detected by the device.

The detection algorithm masks portions of the data deemed unreliable, such as short periods of dramatic rapid signal changes that indicate a watering event like rain or irrigation, or voltage measurements so low (due to locally saturated water) as to cause low-resolution data and consequently unreliable R-C calculations. Additionally, at times, issues with the base station or individual root trackers result in temporary down time, whereby RootTracker data are simply unavailable for analysis. Collectively, these missing data affect a RootTracker’s daily fractional uptime, $U_{t}$. Thus, to calculate a RootTracker’s daily root detection rate, $r_{t}$, we normalize by the RootTracker’s $U_{t}$ for that day:

(3) $$r_{t}=\;\frac{n_{t}}{U_{t}}$$

where $n_{t}$ is the number roots detected for an RootTracker in a given day, t. Days with $U_{t}\leq 0.04$ were treated as $U_{t}=0$ and were ignored to avoid unrealistic values of $r_{t}$ due to limited available data. We calculated daily growth rate by day at a specific electrode depth, $r_{td}$, by aggregating all detections of a RootTracker recorded at a specific electrode depth, d, (from any of the RootTracker’s 12 paddles), $n_{td}$, and normalizing by the relative amount of data available at that depth for that day $U_{td}$.

(4) $$r_{td}=\;\frac{n_{td}}{U_{td}}$$

We applied a low-pass Butterworth filter to the data presented in plots of mean growth rates over time, ${\overset{-}{r}}_{t}$ (and their respective 2× standard errors) with a normalized cutoff frequency of 0.4. We similarly applied a low pass filter to the data presented in the heatmaps of mean growth by depth and time, ${\overset{-}{r}}_{td}$, filtering first by depth, then by time, both passes with cutoff filters of 0.4. The same filtering was applied to plots of mean time-averaged growth rates by depth, ${\overset{-}{R}}_{d}$ (and their respective 2× standard errors). To calculate the cumulative roots detected over time, $c_{t}\;$we integrate aggregate $r_{t}$ over time:

(5) $$c_{t}=\;\sum_{i=0}^{i=t}r_{i}$$

We calculate time-averaged growth rates as follows:

(6) $$R=\;\frac{\sum_{t=a}^{t=b}r_{t}}{b-a}$$

where a and b are the start and end days of the time-averaged period, respectively. Similarly, we calculate time-averaged growth rates at specific electrode depths as follows:

(7) $$R=\;\frac{\sum_{t=a}^{t=b}r_{td}}{b-a}$$

In box and whisker plots of R for different RootTrackers of a specific group, such as seen in Figures 3D or 6A, we excluded RootTrackers from the distribution that had minimal data available for their time average. Specifically, we removed RootTrackers for which >50% of the days of the time-averaging period had no data available ($U_{t}=0$) or where the span of days with data available for calculation was <80% of the period date range.

For all *t* tests calculated in the article, we did not make an assumption of equal variance between the two groups. Thus, we used the Welch two-sample *t* test (one-sided). Calculating Cohen’s *d* effect size also involved using the average variance of the two groups. The two-way interaction analysis of variance (ANOVA) test performed in the R programming environment to compare the drought response of the different hybrids in Trial 1 had the following model: Rate = Treatment + Hybrid + Treatment*Hybrid.

## Results

### The RootTracker uses impedance touch sensing to detect roots

**Figure 1:**
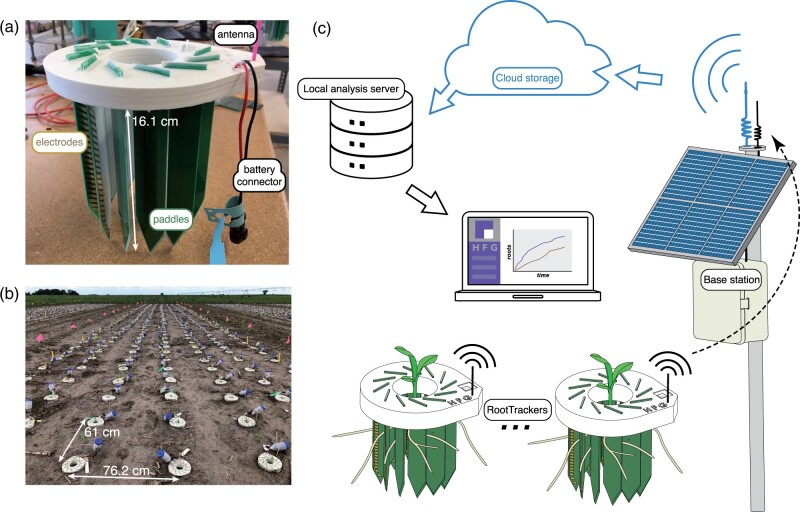
The RootTracker system. The (A) RootTracker consists of 22 electrodes on each of 12 paddles for detecting roots. B, Hundreds of RootTrackers in a typical field installation. C, Diagram illustrating how multiple RootTrackers in a field communicate raw sensor data via radio transmission to a central base station, which sends the data to cloud-based servers, where the data can be analyzed on local servers for extracting root detections.

The RootTracker is made up of 12 circuit board “paddles”, which are arranged in a cylindrical, window shutter-like fashion. Paddles have V-shaped ends to facilitate entry into the soil. Each paddle uses a vertical array of 22 equally spaced impedance sensing electrodes to detect roots (Figure 1A). The electrodes are connected to electronics on a ring-shaped circuit board, which is covered in urethane for mechanical strength and water-proofing. RootTrackers can be installed in field soil (Figure 1B) using hammers or hydraulic presses. Once installed in soil, the electrodes range in depth from 1.9 to 16.1 cm. Seeds can either be sown in the center of the device after installation, or the RootTracker can be centered and inserted over a growing seedling.

After the RootTracker is powered (via a 4-AA battery pack), it takes multiple raw voltage measurements at each electrode and communicates this data wirelessly to a central base station, which then uploads the data to a cloud-based server (Figure 1C). Once downloaded locally, raw voltage data (Supplemental Figure S1A) is processed and converted into electrical capacitance and resistance signals (Supplemental Figure S1B, see “Materials and Methods” for more details). We identified signature fluctuations in the resistance/capacitance space that indicate root growth activity near the sensors once signal changes have been normalized across all electrodes. In this way, fluctuations are evaluated to detect roots. Because all RootTrackers are oriented in the same way and the planting depth is consistent across the field, RootTracker detections capture the initial depth and direction of a root’s growth. Since these detections are timestamped, root growth rates can be computed.

### RootTracker measurements correlate with root image analysis

**Figure 2:**
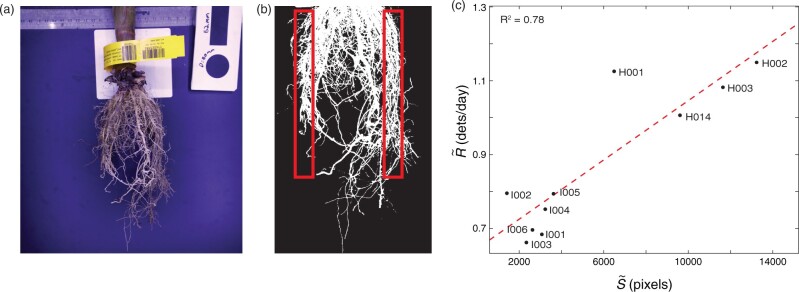
Shovelomics comparison in Trial 1. A, Sample photograph of root system excavated from a RootTracker. B, Image segmenting for root pixels. Root pixels located in the region that would interact with RootTracker detectors (red boxes) were counted for each plant. C, Median daily root detection rate time-averaged across the entire trial, $\tilde{R}$, grouped by genotype, versus median shovelomics image root pixels, $\tilde{S}$, grouped by genotype. Correlation between RootTracker detections and shovelomics root characterization: $R^{2}$ = 0.78.

**Figure 3:**
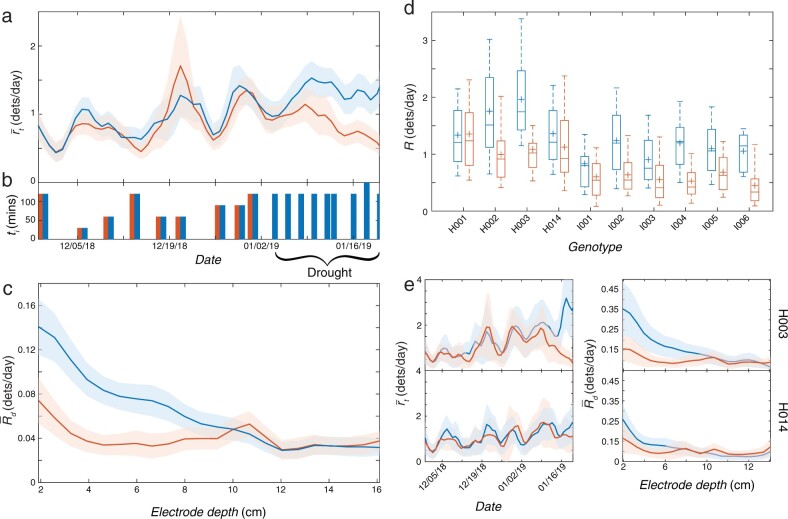
Trial 1: Response to imposed drought. A, Comparison of root detections between well-watered plants (blue) and drought treatment plants (orange) of smoothed mean daily root detection rate over time, ${\overset{-}{r}}_{t}$. B, Duration, $t_{i}$, of each irrigation event during the trial for each treatment. C, Comparison of root detections between well-watered plants (blue) and drought treatment plants (orange) of the smoothed mean of the per-electrode-depth daily root detection rate, time-averaged across the drought period (January 4, 2019 to January 19, 2019), ${\overset{-}{R}}_{d}$. All shaded regions indicate ±2 standard errors from the mean. D, Box and whisker distributions of time-averaged daily root detection rate, R, during the drought period, separated by genotype and treatment (blue, well-watered; orange, drought). Top and bottom of box indicate 25 and 75 percentiles of RootTrackers; horizontal line in box is median; cross is mean, and whiskers are 9 and 91 percentiles. E, Well-watered (blue) and drought (orange) treatment comparison of ${\overset{-}{r}}_{t}$ (left) and ${\overset{-}{R}}_{d}$ during the drought period (right) for two hybrids (H003, top, and H014, bottom) with variable relative responses to drought.

To validate measurements made with the RootTracker, we performed a ground-truth procedure by comparing RootTracker detections with analyzed images of excavated roots (Figure 2), similar to shovelomics (Trachsel et al., 2011). Data for the ground truth procedure were extracted from a drought study (Trial 1) that examined the response in root growth of different maize varieties to water deficits. We installed 881 RootTrackers in alluvial soil at Massai Agricultural Services field station in Rancagua, Chile under drip irrigation. We selected 10 genotypes (four hybrids and six inbreds) and subjected them to two watering treatments, well-watered and drought. Plots under the well-watered treatment followed a schedule of periodic irrigation for 53 d (Figure 3B). Plots under the drought treatment had the same irrigation schedule until day 36, when the water was shut off for the remainder of the experiment (a 16-d period). After Trial 1, roots were excavated, washed, imaged, and analyzed according to the protocol in the “Materials and methods”.

We used the excavated root images to measure the total pixels that were located in the same region of soil as the RootTracker detectors would have been. Known dimensions of the RootTracker, consistent placement of the root crown in the frame of the image, and a known scale for pixels were used to identify this region (red rectangles in Figure 2B, see “Materials and methods” for more details). We found a strong correlation between our proxy for root mass—daily root detection rate time-averaged across the entire trial—versus root pixels in the images (Figure 2C), verifying that the RootTracker platform and shovelomics produce similar measurements. Compared to destructive root image analysis, the RootTracker is advantageous in that it is able to monitor root growth noninvasively, thus providing information about root system growth and its response to environmental factors, not just a measure of its final architecture.

Using the same shovelomics procedure, we performed an additional ground truth comparative analysis of sorghum (*Sorghum bicolor*) roots excavated in August 2019 at the conclusion of a RootTracker trial performed in Darlington, South Carolina, which consisted of sandy soil (as compared to the alluvial soil in Rancagua, Chile), and found a similarly strong correlation (Supplemental Figure S2, see “Materials and methods” for more details on this trial).

### Root growth rate is reduced in response to water deficit

Grouping all plants in Trial 1 by watering treatment, RootTracker detection analysis indicated that plants responded to the imposed water deficit by rapidly reducing root growth. The average daily root detection rate (${\overset{-}{r}}_{t}$) under both treatments was approximately the same until the water deficit was imposed (Figure 3A). The mean time-averaged daily root detection rate, $\overset{-}{R}$, for plants subjected to the water deficit decreased to 0.81 root detections per day, ∼36% fewer than the well-watered plants during the same time period (one-sided *t* test, $t_{df=572.8}=$ 8.4, P ≤2.2e−16, Cohen’s *d* = 0.70, 95% confidence lower bound: 0.37). When analyzed by electrode depth during the water deficit, drought treatment plants exhibited lower average daily growth rates than well-watered plants in shallower soil strata, while at deeper soil levels, there was little difference in the number of roots detected (Figure 3C). These results indicate fewer roots grew near the surface, likely owing to drying of the soil surface after the irrigation was shut off.

To examine genotypic differences in response to the water deficit, we analyzed root growth of each genotype separately (Figure 3D). Within our collection of four hybrids, we found an interaction between hybrid and watering treatment (interaction two-way ANOVA, P = 0.003), indicating variation in drought response by hybrid. Two genotypes (H003 and H014; Figure 3E) highlight this divergent behavior. During the time of the water deficit, H014 exhibited little change in daily root detection rates. Surprisingly, H003 plants under drought conditions had similar daily root detection rates to those of H014. Thus, rather than having a decreased negative response to drought, H014 appears to have a decreased positive response to receiving water.

### Root system dynamics may contribute to priming

**Figure 4:**
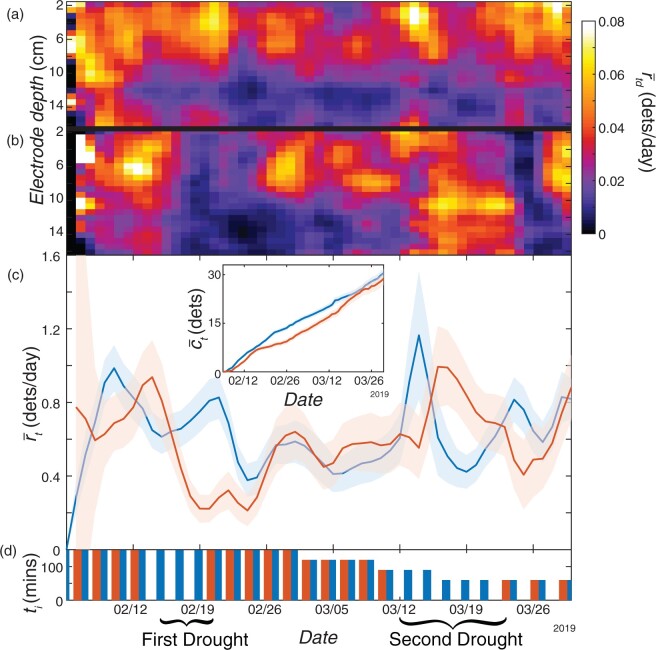
Trial 2: Priming response to early drought. Comparison between (A) well-watered plants and (B) water-limited plants, of smoothed mean daily root detection rate over time and electrode depth, ${\overset{-}{r}}_{td}$. C, Comparison between well-watered plants (blue) and water-limited plants (orange) of smoothed mean daily root detection rate over time, ${\overset{-}{r}}_{t}$, and (c inset) mean cumulative root detections over time, ${\overset{-}{c}}_{t}$. All shaded regions indicate ±2 standard errors from the mean. D, Duration, $t_{i}$, of each irrigation event during the trial for each treatment.

Mild drought stress early in the growth of wheat (*Triticum aestivum L*.) can cause a “priming effect”, whereby the plant responds to an early stress by better tolerating later episodes of stress, such as reduced water availability (Wang et al., 2014). If a similar priming effect occurs in maize, we hypothesized that root system dynamics could play a role in this response. To test this hypothesis, we installed 721 RootTrackers in Rancagua, Chile divided among 12 hybrid genotypes and two irrigation treatments: well-watered, and water-limited. In the latter irrigation treatment, we imposed two separate water deficits: the first starting 9 d after planting and lasting 6 d, the second starting 35 d after planting and lasting 11 d (Figure 4D).

During the first drought period, we observed a rapid decrease in root growth rate, similar to Trial 1 (Figure 4C). Then, remarkably, the average daily rate of detection during the second water deficit between March 15, 2019 and March 22, 2019 rose by 0.88 roots per day—148% higher than well-watered plants (one-sided *t* test, $t_{\mathrm{df}\;=\;327.04}=$ 7.4, P = 7.84e−13, Cohen’s *d* = 0.74, 95% confidence lower bound: 0.41). Although this coincided with the second drought, evidence presented in the next section indicates that the response may be primarily a delayed reaction to the first water deficit. The comparison of cumulative root growth over time suggests that the subsequent increased root detections allowed these plants to approach the average cumulative detections of well-watered plants (Figure 4C, inset). Additionally, during the same time period that we observed overall increased root growth, water-limited root detections were concentrated at the deeper electrodes, whereas the well-watered root detections were concentrated at more shallow electrodes (Figure 4, A and B; see Supplemental Figure S3 for complementary heat maps of standard error). From March 15, 2019 to March 22, 2019, we observed a significant increase in average daily growth rate at the deeper half of electrodes in water-limited plants as compared to well-watered plants (one-sided *t* test, $t_{\mathrm{df}\;=\;290.84}=$ 8.7, *P* < 2.2e−16, Cohen’s *d* = 0.85, 95% confidence lower bound: 0.31). The mean growth rate in the deeper half in water-limited plants was 0.50 roots per day, which is 319% greater than that in well-watered plants. These results indicate that an early water deficit can promote more root growth in deeper soil strata later in the growing season, even during a second imposed drought.

### The priming response is independent of a second water deficit

**Figure 5:**
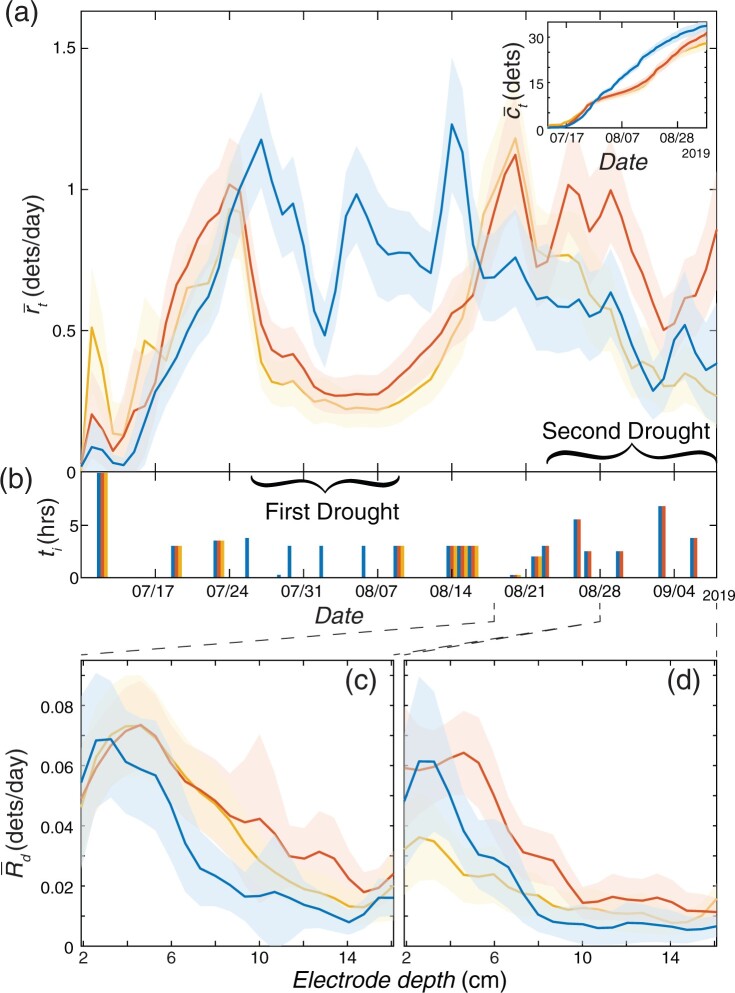
Trial 3: Priming response to early drought. Comparison between well-watered plants, (blue), single drought plants (orange), and double drought (yellow), of (A) smoothed mean daily root detection rate over time, ${\overset{-}{r}}_{t}$, and (A, inset) mean cumulative root detections over time, ${\overset{-}{c}}_{t}$. B, Duration, $t_{i}$, of each irrigation event during the trial for each treatment. Comparison between well-watered plants (blue), single drought plants (orange), and double drought (yellow), of the smoothed mean of the time-averaged, per-electrode-depth daily root detection rate, ${\overset{-}{R}}_{d}$, from (C) August 18, 2019 to August 28, 2019 and (D) August 28, 2019 to September 8, 2019. All shaded regions indicate ±2 standard errors from the mean.

To determine if a second water deficit is required to induce the increased root growth observed in Trial 2, we conducted a follow-up experiment at the KARE Center located in Parlier, California during the summer of 2019. Soil and climate conditions resembled those found at the test site in Chile. We used 1,457 RootTrackers divided among 10 hybrid genotypes (six of which were included in Trial 2) and three irrigation treatments. The well-watered treatment followed the irrigation schedule illustrated in Figure 5B. The furrows between the planted rows were drip irrigated to produce consistent water diffusion in the soil. The single drought treatment consisted only of an early water deficit (14-d water shutoff starting 16 d after planting). The double drought treatment consisted of the same early drought, as well as a second drought (16-d water shut-off starting 44 d after planting), similar to the water-limited treatment in Trial 2.

As in Trials 1 and 2, we observed a rapid decrease in the average root detection rate during the first drought period for single and double drought treatment plants as compared to well-watered plants (Figure 5A, one-sided *t* test, $t_{\mathrm{df}\;=\;467.31}=$ 16.2, *P* <2.2e−16, Cohen’s *d* = 1.1, 95% confidence lower bound: 0.49). The average rate for single and double drought plants during the first drought period was 0.31 root detections per day, 64% less than that of well-watered plants during the same time period. See Supplemental Figure S4 for per-genotype responses to drought. Following the first drought, similar to water-limited plants in Trial 2, double drought plants in Trial 3 also temporarily exhibited increased detection rates relative to well-watered plants. The average daily root detection rate of double drought plants for a period leading into the second drought (August 18, 2019 to August 28, 2019) was 0.87 roots per day—40% greater than well-watered plants (one-sided *t* test, $t_{\mathrm{df}\;=\;572.6}=$ 4.0, *P*=2.98e−5, Cohen’s *d* = 0.33, 95% confidence lower bound: 0.15). However, the detection rate of the double drought plants began to decrease once the second drought began. In contrast, single drought plants exhibited an increase in detection rates beginning at about the same time as was observed for double drought plants and sustained increased growth to the end of the trial. The average daily root detection rate of single drought plants from August 18, 2019 to September 8, 2019 was 0.80 roots per day, 46% greater than well-watered plants (one-sided *t* test, $t_{\mathrm{df}\;=\;643.8}=$ 5.9, *P*=3.29e−9, Cohen’s *d* = 0.46, 95% confidence lower bound: 0.18). In the time following the end of the first drought, we found that the cumulative detections of single drought plants approached those of well-watered plants (Figure 5A inset). In contrast, and unlike in Trial 2, double drought plants in Trial 3 were unable to approach the average cumulative root detections of well-watered plants.

While not as pronounced as in Trial 2, we similarly observed that single and double drought plants exhibited greater rates of root detections at the deeper electrodes relative to well-watered plants following the first drought (Figure 5C). The average daily root detection rate of single and double drought plants in the deeper half of the electrodes from August 18, 2019 to August 28, 2019 was 0.24 roots/day, 69% greater than well-watered plants (one-sided *t* test, $t_{\mathrm{df}\;=\;577.69}=$ 5.7, P=1.19e−8, Cohen’s *d* = 0.39, 95% confidence lower bound: 0.07). Strikingly, even when the overall average detection rate of double drought plants was reduced to similar levels as well-watered plants following the initial increase in root growth rate, double drought plants still exhibited more detections at the deeper electrodes than well-watered plants (Figure 5D). The average daily root detection rate of double drought plants in the deeper half of electrodes from August 28, 2019 to September 8, 2019 was 0.12 roots/day, 79% greater than well-watered plants (one-sided *t* test, $t_{\mathrm{df}\;=\;525.52}=$ 3.2, *P* = 0.0006, Cohen’s *d* = 0.26, 95% confidence lower bound: 0.03). Consequently, the double drought root detection rates during this time period were also marked by fewer detections in the shallow region. In summary, an early water deficit induces increased root growth in maize seedlings at somewhat deeper soil levels later in the growing season.

### The RootTracker identifies a wide array of root phenotypes present in maize

**Figure 6:**
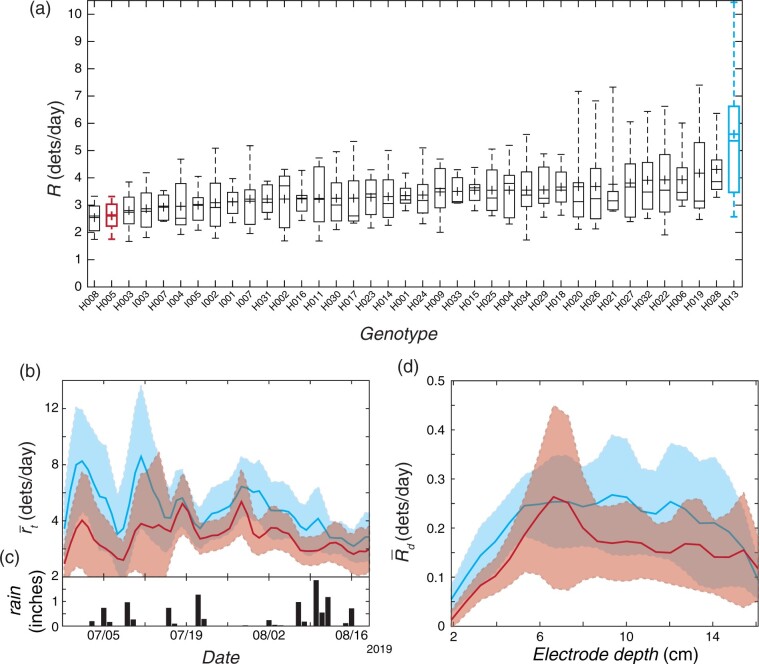
Trial 4: Comparison of maize genotypes in Midwest fields. A, Box and whisker distributions of daily root detection rates time-averaged across the entire trial, R, separated by genotype. Top and bottom of box indicate 25 and 75 percentile of RootTrackers, horizontal line in box is median, cross is mean, and whiskers are 9 and 91 percentiles. B, Comparison of root detections between plants of genotype H005 (red) and H013 (blue) of smoothed mean daily root detection rate over time, ${\overset{-}{r}}_{t}$. C, Inches of rain for each day received at Aurora, NE by station GHCND:US10hami004 (data provided by the National Oceanic and Atmospheric Administration (NOAA) National Climatic Data Center (NCDC) National Centers for Environmental Information, Asheville North Carolina from their website at ncdc.noaa.gov, Menne et al., 2012a). D, Comparison of root detections between plants of genotype H005 (red) and H013 (blue) of smoothed mean of the per-electrode-depth daily root detection rate, time-averaged across the entire trial, ${\overset{-}{R}}_{d}$. Shaded regions indicate ±2 standard errors from the mean.

Previous analyses using shovelomics and gel-based growth media indicated that root phenotypes differ across a maize nested association mapping population (Hauck et al., 2015; Zurek et al., 2015). However, there is very little available data on root systems of commercial maize hybrids. To gain insight into the level of natural variation in root phenotypes among commercial maize hybrids, we used 1,482 RootTrackers to monitor root growth of 32 hybrids with high yield potential as well as six inbred lines. The trial was performed at Real Farm Research in Aurora, Nebraska in a loamy silt soil where maize and soybeans (*Glycine max*) are typically grown. Inspection of root growth records over 55 d demonstrated that there were significant differences in root detection rates (Figure 6, one-way ANOVA, F(37) = 1.93, *P* = 0.0013). Two contrasting genotypes (H005 and H013) highlight these differences by both time and depth (Figure 6, B–D). These results provide evidence that a broad range of root phenotypes is present in the germplasm of modern maize hybrids.

## Discussion

The RootTracker enables direct measurement of root system dynamics in soil. Current methods to measure root growth in the field are either destructive (e.g. shovelomics) or sample a small and localized subset of roots (e.g. mini-rhizotrons). We have described a sensor-based technology able to monitor root growth over time in field conditions. In addition to providing a scalable platform for root phenotyping, communication of raw sensor data from remote locations is facilitated by relatively small file sizes (∼1 kB per RootTracker every 5 min) as compared to image-based modalities, and subsequent conversion of raw data to root detections occurs offline.

The RootTracker technology is designed to detect roots that emerge from the main body of the plant, e.g. crown and seminal roots as well as lateral roots that form from those. Detection of these roots over time provides a measure of the rate of root growth. This information is particularly useful in assessing seedling establishment and response to environmental perturbations such as water deficits. Knowledge of the depth of detection allows inference of the angle of root growth, at least for crown and seminal roots whose origin is close to the original location of the seed. Other metrics available from the RootTracker are orientation of growth and time of day of maximum root growth. None of these measures is likely to encompass all roots made by the plant. Rather, they allow comparisons between genotypes, environments and management practices with the goal of identifying how they impact root system dynamics. With the current sensor device, we cannot directly measure root growth beyond the depth of the paddles. Future iterations could use longer paddles to access deeper soil strata.

A strong correlation exists between plants with deeper roots and increased tolerance for water deficits (Lynch, 2013; Li et al., 2019). However, there is little knowledge as to how roots temporally and spatially respond to water deficits in the field. Our results clearly show a rapid reduction in root growth when irrigation is shut off with a more dramatic response in the shallow soil strata. This suggests that maize plants can modulate root growth in response to small differences in soil moisture, opening the possibility of breeding plants with root systems optimized to respond to drought by growing deeper when a water deficit is detected. The RootTracker provides an opportunity to quantitatively characterize the response phenotypes in a wide array of different water availability scenarios (with variations in timing relative to plant development, duration, as well as severity) in not only a controlled irrigation context, but also in the context of rain events such as in Trial 4, where daily root detection rates suggest that the plants exhibited growth rate fluctuations that coincided with rain events early in the trial (Figure 6, B and C).

The first few weeks of growth are important for the viability and robustness of row crops. An early exposure to abiotic stress known as “priming” has been shown to provide a measure of protection against later stresses in wheat. Our results suggest that the same may be true of maize. Root growth monitoring by the RootTracker indicated that imposing an early water deficit resulted in maize plants with more and deeper roots later in the growing season. In Trials 2 and 3, we identified increased root growth subsequent to an early drought at a time coinciding with a second imposed drought. This increased growth also occurred in plants that were only subjected to a first priming drought. Furthermore, the single drought plants sustained increased root growth for a longer period of time as compared with plants that were subjected to two droughts. This suggests that the increased growth rate was primarily a priming effect resulting from the earlier water deficit, and the timing of the second drought in Trials 2 and 3 was coincidental relative to the timing of the observed augmented growth rates.

Beyond aggregate root growth, monitoring roots with the RootTracker revealed how plants may be able to simultaneously respond both to current environmental conditions as well as stresses that occurred at earlier growth stages. For example, toward the end of the second drought in Trial 3, when daily root growth of the double drought plants was similar to the well-watered plants, the double drought plants exhibited relatively greater amounts of roots in the deeper portion of the RootTracker and fewer shallow roots. This suggests a trade-off as to how to allocate new roots in search of water. Furthermore, while the single drought plants in Trial 3 primarily grew roots in the shallow soil strata toward the end of the experiment, they still exhibited greater root growth at the deeper electrodes than the well-watered plants. This suggests that plants can modulate the depth of new roots during and after imposed droughts.

It is possible that root systems for commercial hybrids have been optimized during the intense selection for increased yield over the last century. Our data from a Midwestern field suggests that there remains a substantial amount of natural variation for root phenotypes in elite maize lines. A plausible reason is that most maize breeding has been performed under nutrient and water-replete conditions presenting minimal selection pressure on root growth. The presence of alleles in the genome of elite cultivars for different root phenotypes provides an exciting opportunity to identify and breed for plants that are optimized for specific environments and that mitigate greenhouse gas emissions.

## Conclusion

We describe a proof-of-concept use for the RootTracker in identifying root phenotypes in maize. The platform can be adapted to other row crops, either in its present form or with modifications in its form factor. Moreover, it can be used both in the field and in controlled environment settings. Thus, the RootTracker platform provides the opportunity to discover how roots grow in different soils and respond to different stimuli.

## Supplemental data

The following materials are available in the online version of this article.

**Supplemental Figure** **S1**. Sample raw electrode signal over time from a Version 2 RootTracker between the dates of February 15, and February 25, 2019 in Trial 2.

**Supplemental Figure S2**. Shovelomics comparison of Version 2 RootTrackers from a field trial of sorghum grown in South Carolina.

**Supplemental Figure S3**. Standard error by depth and time of root detections in Trial 2.

**Supplemental Figure S4**. Per-genotype responses to drought in Trials 2 and 3.

**Supplemental Figure S5**. Trial 1 field map (Massai Agricultural Services, Rancagua, Chile, 2018–2019).

**Supplemental Figure S6**. Trial 2 field map (Massai Agricultural Services, Rancagua, Chile, 2019).

**Supplemental Figure S7**. Trial 3 field map (Kearney Agricultural Research and Extension (KARE) Center located in Parlier, California, 2019).

**Supplemental Figure S8**. Trial 4 field map (Real Farm Research, Aurora, Nebraska, 2019).

**Supplemental Figure S9**. Analysis of soil in Trials 1 and 2.

**Supplemental Figure S10**. Shovelomics comparison of different RootTracker versions.

**Supplemental Table S1**. Summary of RootTracker trials.

**Supplemental Table S2**. Weather data by month and location.

**Supplemental Table S3**. Number of RootTrackers (N) by genotype and treatment for each trial.

**Supplemental Data Set S1**. List of RootTrackers in Trial 1 and their corresponding treatment, genotype, hardware version and location on the field.

**Supplemental Data Set S2**. List of RootTrackers in Trial 2 and their corresponding, treatment, genotype, hardware version and location on the field.

**Supplemental Data Set S3**. List of RootTrackers in Trial 3 and their corresponding, treatment, genotype and location on the field.

**Supplemental Data Set S4**. List of RootTrackers in Trial 4 and their corresponding genotype and location on the field.

## Supplementary Material

kiab352_Supplementary_Data

## Notes

P.N.B. and J.B.W. conceived the idea of a simple in-field device for detecting roots. J.J.A, M.M., L.J., J.B.W., J.T., and J.L.E. developed the RootTracker prototype. J.J.A, M.M., L.J., J.T., J.L.E., D.W., F.O., and E.R. performed research and development tests on the root sensing technology. D.W., F.O. fabricated the root sensing devices used in trials. J.J.A. and J.B.W. analyzed the data. J.J.A, P.N.B., and J.B.W. wrote the manuscript. S.F. maintained Trial 3, collected notes on plants, and wrote the methods for Trial 3. R.G., J.B.W., L.J., E.R., and J.J.A conceived the trials. R.G. designed the field layouts for and oversaw trials. P.N.B. and J.B.W. provided direction and supervised the project. F.O. and E.R. collected excavated roots from the sorghum (Sorghum bicolor) trial, and F.O. performed the image analysis.

The author responsible for distribution of materials integral to the findings presented in this article in accordance with the policy described in the Instructions for Authors (https://academic.oup.com/plphys/pages/general-instructions) is: Jeffrey J. Aguilar (jeff.aguilar@hifidelitygenetics.com).
