# Supplementary material for: Capturing in-field root system dynamics with RootTracker
Source: Plant Physiol. 2021 Jul 27;187(3):1117–30. doi: 10.1093/plphys/kiab352 (PMC8566282; doi:10.1093/plphys/kiab352)
Supplement: kiab352_Supplementary_Data [file kiab352_supplementary_data.zip › Supplemental Data.docx]

Supplemental Figure S1. Sample raw electrode signal over time from a Version 2 RootTracker between the dates of February 15, and February 25, 2019 in Trial 2. (a) Raw voltage data of sample electrode collected when the electrode was charged for one microsecond. (b) Scatter plot of electrical resistance and capacitance measured for the same electrode as in (a) during the same time period. Resistance and capacitance are calculated using both one microsecond charge time and 255 microsecond charge time voltage measurements.

Supplemental Figure S2. Shovelomics comparison of Version 2 RootTrackers from a field trial of sorghum grown in South Carolina. Median daily root detection rate time-averaged across the entire trial, $\tilde{R}$, grouped by genotype, versus median shovelomics image root pixels, $\tilde{S}$, grouped by genotype. S1-S11 are sorghum genotypes. C is a genotype of maize.


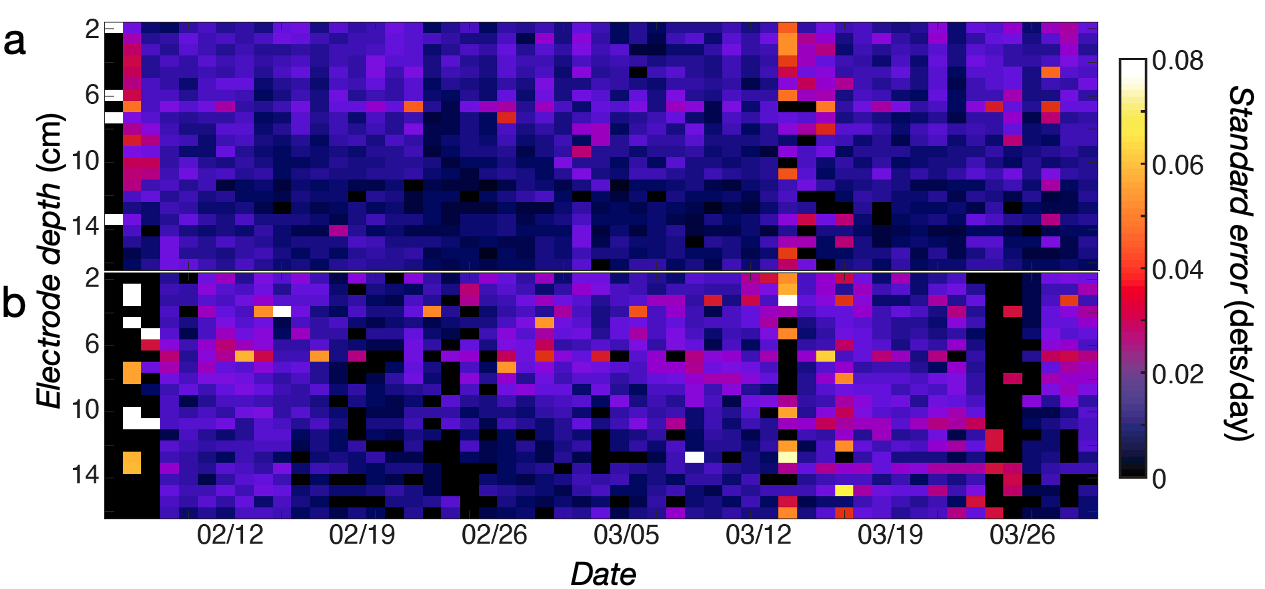


Supplemental Figure S3. Standard error by depth and time of root detections in Trial 2. Heatmaps of standard error for mean daily root detection rate over time and electrode depth, $\bar{r}_{td}$ for (a) well-watered plants, and (b) water-limited plants in Trial 2.

Supplemental Figure S4. Per-genotype responses to drought in Trials 2 and 3. Box and whisker distributions of time-averaged daily root detection rates, $R$, separated by genotype and treatment, time-averaged during: (a) a period (2/16/2019 - 2/22/2019) overlapping with the early drought in Trial 2, (b) part of the late drought in Trial 2 (3/15/19 - 3/22/19), (c) the early drought in Trial 3 (7/26/2019 - 8/9/2019), (d) a period (8/18/2019 - 8/28/2019) overlapping with the beginning of the second drought in Trial 3, and (e) a period (8/28/2019 - 9/8/2019) overlapping with the end of the second drought in Trial 3. For Trial 2 (a,b), the color code is as follows: well-watered: blue; water-limited: orange. For Trial 3 (c-e), the color code is as follows: well-watered: blue; single drought: orange; double drought: yellow. Top and bottom of box indicate 25 and 75 percentiles of RootTrackers; horizontal line in box is median; cross is mean, and whiskers are 9 and 91 percentiles.


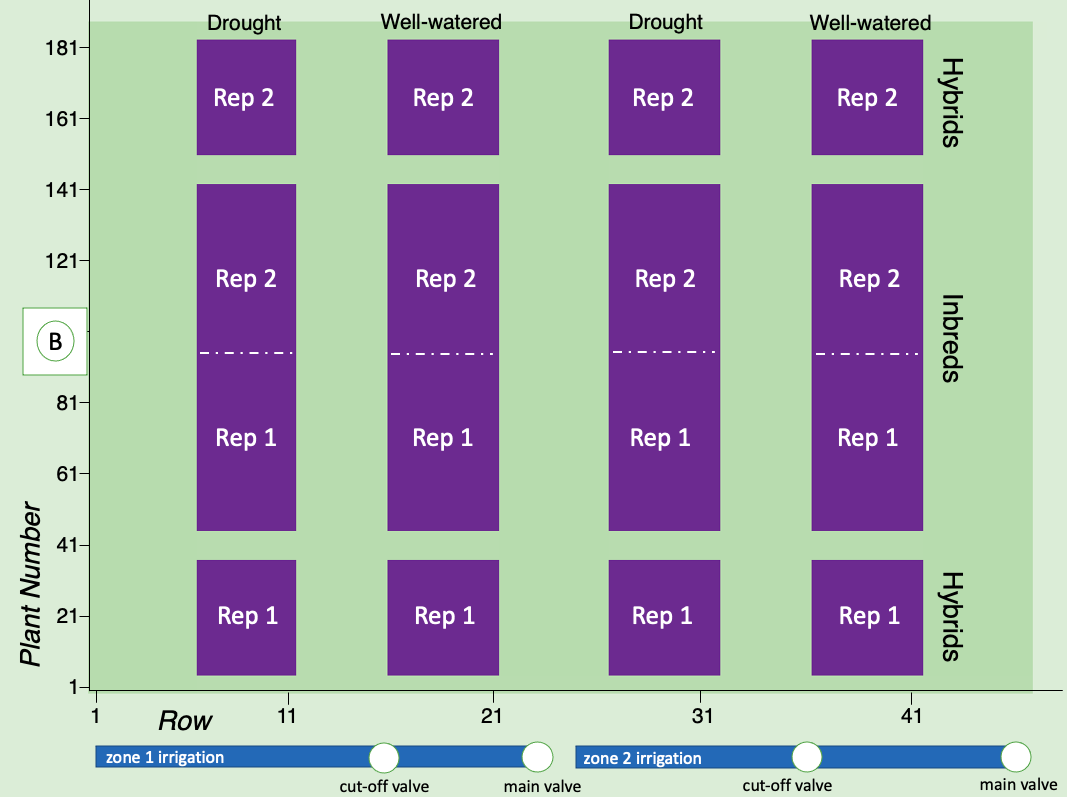


Supplemental Figure S5. Trial 1 field map (Massai Agricultural Services, Rancagua, Chile, 2018-19). Rows were spaced 30” apart and plants within rows were 1’ apart. The base stations (B) were located to the left of the field half-way down the rows. Two irrigation treatments (drought and well-watered) were applied in blocks 5 rows wide and 173 feet long (purple), separated by 5 or 6 rows of border plants (dark green). The experiment included two reps of each treatment, and 10 genotypes (4 hybrids and 6 inbred lines) were tested across each treatment block in two reps. To minimize neighboring canopy and treatment effects, the field was laid out in a split plot design with hybrids and inbreds blocked within drought and well-watered treatment blocks. Genotypes were randomized within blocking factors and planted in plots 8 plants long by 5 rows wide. Drip lines were attached to one of two main water lines (irrigation zones 1 and 2). Cutoff valves allowed for a drought and a well-watered section along each main water line. Version 2 RootTrackers were primarily located in the right two treatment blocks, and version 1 RootTrackers were installed primarily in the left two treatment blocks. See Supplemental Dataset S1 for a list of RootTrackers and their corresponding treatment, genotype, hardware version and location on the field.


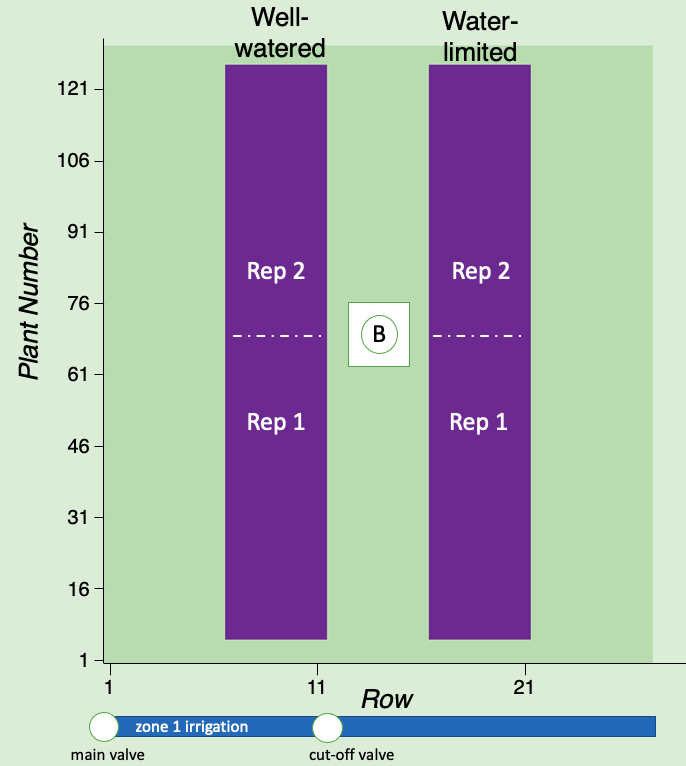


Supplemental Figure S6. Trial 2 field map (Massai Agricultural Services, Rancagua, Chile, 2019). Rows were spaced 30” apart and plants within rows were 1’ apart. The base stations were located at the center of the field. Treatments (well-watered and water-limited) were applied in separate blocks 5 rows wide and 120 feet long (purple), separated by 6 rows of border plants (dark green). All plants in treatment rows (purple) were in RootTrackers. Drip lines were attached to a main water line (irrigation zone 1) with a cutoff valve that allowed for separate well-watered and water-limited sections. The experiment included 12 hybrid genotypes. Genotypes were randomized within treatment blocks, planted in plots 5 plants long by 5 rows wide, and tested across each treatment block in two reps. Version 2 RootTrackers were primarily installed at bottom half of the water-limited treatment block. See Supplemental Dataset S2 for a list of RootTrackers and their corresponding, treatment, genotype, hardware version and location on the field.


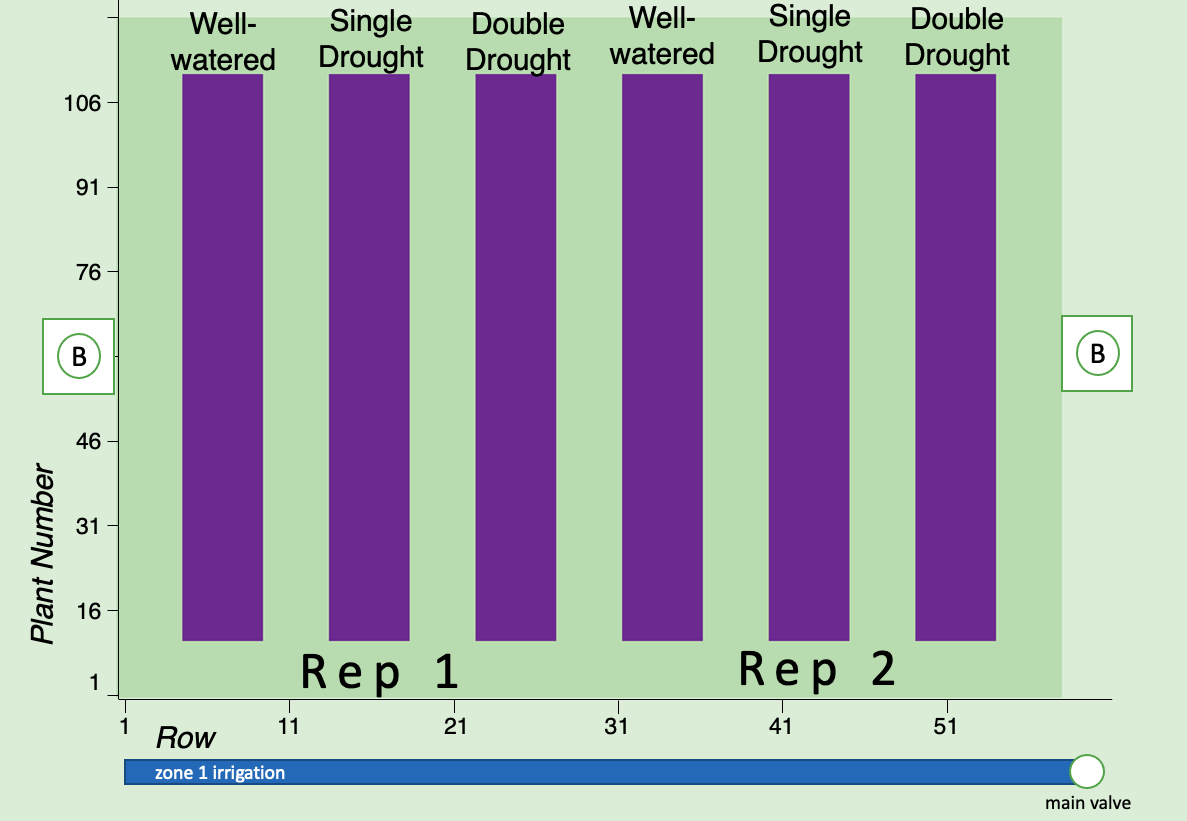


Supplemental Figure S7. Trial 3 field map (Kearney Agricultural Research and Extension (KARE) Center located in Parlier, California, 2019). Rows were spaced 30” apart and plants within rows were 1’ apart. The base stations were located half-way down the rows on either side of the field. Treatments (well-watered, single drought, and double drought) were applied in separate blocks 5 rows wide and 99 feet long (purple), separated by 4 rows of border plants (dark green). Treatment rows (purple) contained RootTrackers for every other plant. Drip lines were attached to a main water line (irrigation zone 1) with a cutoff valve that allowed for separate well-watered and water-limited sections. Each drip line was individually controlled by a manual valve. The experiment included 10 hybrid genotypes. Genotypes were randomized within treatment blocks, planted in plots 10 plants long by 5 rows wide, and tested across each treatment block in two reps. See Supplemental Dataset S3 for a list of RootTrackers and their corresponding, treatment, genotype and location on the field.


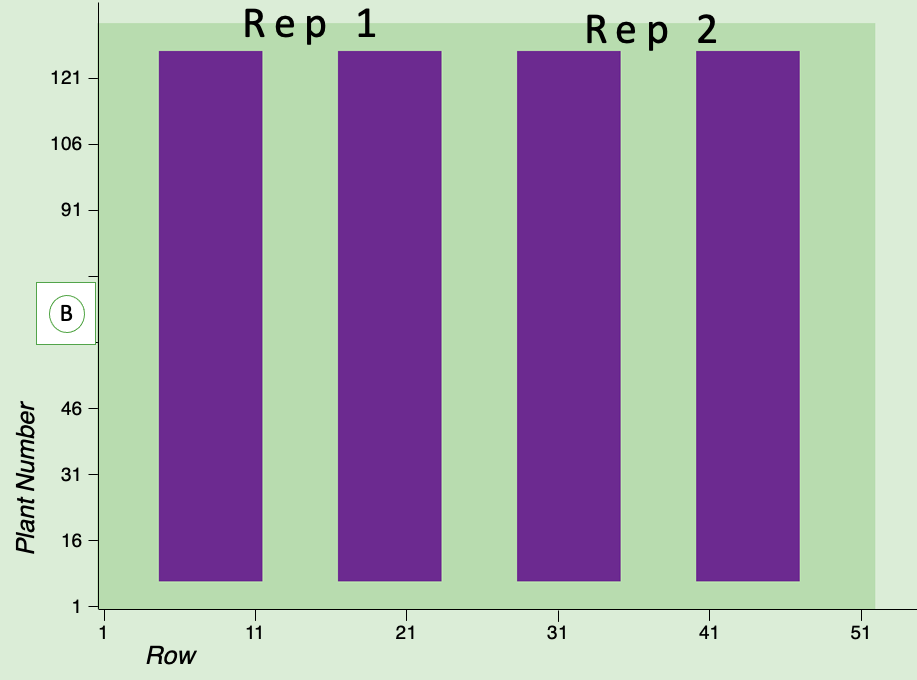


Supplemental Figure S8. Trial 4 field map (Real Farm Research, Aurora, Nebraska, 2019). Rows were spaced 30” apart and plants within rows were 1’ apart. The base stations were located to the left of the field half-way down the rows. Experimental blocks of the field (purple) were 7 rows wide by 114 feet long and were surrounded by 4 or 5 rows of border plants (dark green). Rows in the experimental blocks contained RootTrackers every other plant (i.e. spaced 2 ft apart). Experimental blocks were 7 rows wide and 113 plants long. The experiment included 25 hybrid and 13 inbred genotypes tested in two reps, where the left two experimental blocks contained rep 1 and the right two contained rep 2. To minimize neighboring canopy and treatment effects, hybrids and inbreds were blocked separately. Genotypes were randomized within inbreds and hybrids in both reps and were planted in plots 6 plants long by 7 rows wide. See Supplemental Dataset S4 for a list of RootTrackers and their corresponding genotype and location on the field.

Supplemental Figure S9. Analysis of soil in Trials 1 and 2.


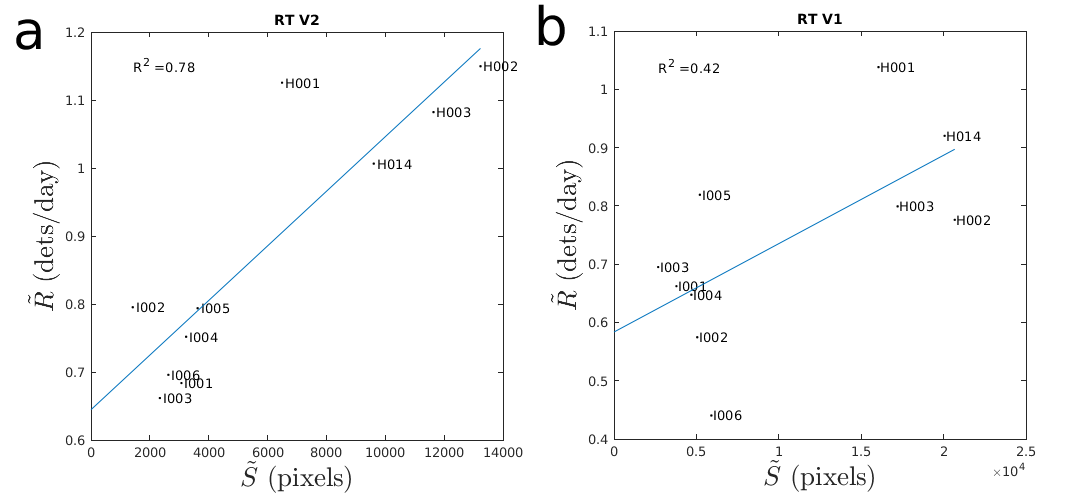


Supplemental Figure S10. Shovelomics comparison of different RootTracker versions. Median daily root detection rate time-averaged across the entire trial, $\tilde{R}$, grouped by genotype, versus median shovelomics image root pixels, $\tilde{S}$, grouped by genotype for (a) Version 2 (V2) RootTrackers vs (b) Version 1 (V1) RootTrackers. The primary difference between the two hardware versions is a change in V2 to the resistance in the voltage divider of the charging circuit allowing for great signal sensitivity in wet soils.

| Trial Name | Location | Trial Period (planting date to end) | Key Time Periods | Treatments | Total # of RootTrackers |
| --- | --- | --- | --- | --- | --- |
| Trial 1 | Rancagua, Chile | 11/29/2018-1/20/2019 | \| drought \| 1/4/2019-1/20/2019 \| \| --- \| --- \| | \| well-watered \| \| --- \| \| drought \| | 1223 (342 Version 1 RootTrackers, 881 Version 2 RootTrackers) |
| Trial 2 | Rancagua, Chile | 2/5/2019-3/30/2019 | \| early drought \| 2/14/2019-2/22/2019 \| \| --- \| --- \| \| late drought \| 3/12/2019-3/23/2019 \| | \| well-watered \| \| --- \| \| water-limited \| | 1154 (433 Version 1 RootTrackers, 721 Version 2 RootTrackers) |
| Trial 3 | Parlier, California | 7/10/2019-9/8/2019 | \| early drought \| 7/26/2019-8/9/2019 \| \| --- \| --- \| \| late drought \| 8/23/2019-9/8/2019 \| | \| well-watered \| \| --- \| \| single drought \| \| double drought \| | 1457 |
| Trial 4 | Aurora, Nebraska | 6/28/2019-8/22/2019 | N/A | N/A | 1482 |
| Sorghum Trial | Darlington, South Carolina | 6/20/2019-8/5/2019 | N/A | N/A | 409 |

Supplemental Table 1. Summary of RootTracker trials.

| Trial | Location | Year | Month | Date Range | Avg Daily Low (° F) | Avg Daily High (° F) | Inches of rain |
| --- | --- | --- | --- | --- | --- | --- | --- |
| Trial 1 | Rancagua, Chile | 2018 | November | 29 to 30 | 50.7 | 82.0 | 0.00 |
|  |  | 2018 | December | 1 to 31 | 52.4 | 86.0 | 0.03 |
|  |  | 2019 | January | 1 to 20 | 54.2 | 84.8 | 0.00 |
| Trial 2 |  | 2019 | February | 5 to 28 | 54.7 | 88.1 | 0.00 |
|  |  | 2019 | March | 1 to 30 | 47.5 | 84.2 | 0.00 |
| Trial 3 | Parlier, California | 2019 | July | 10 to 31 | 66.3 | 97.7 | 0.00 |
|  |  | 2019 | August | 1 to 31 | 65.1 | 96.9 | 0.00 |
|  |  | 2019 | September | 1 to 8 | 65.0 | 95.3 | 0.00 |
| Trial 4 | Aurora, Nebraska | 2019 | June | 28 to 30 | 71.7 | 98.0 | 0.00 |
|  |  | 2019 | July | 1 to 31 | 67.1 | 87.7 | 3.97 |
|  |  | 2019 | August | 1 to 22 | 65.5 | 83.4 | 5.39 |
| Sorghum Trial | Darlington, South Carolina | 2019 | June | 20 to 30 | 68.5 | 91.2 | 0.99 |
|  |  | 2019 | July | 1 to 31 | 70.9 | 91.6 | 6.08 |
|  |  | 2019 | August | 1 to 5 | 69.3 | 88.6 | 0.92 |

Supplemental Table 2. Weather data by month and location. Daily high and low temperatures were averaged according to the date range listed in the Date Range column. Similarly, inches of rain were totaled only during the specified date ranges. Date ranges span the trial durations. Temperature and rain data from for the trials in Rancagua, Chile is provided by a weather station in the nearby area of Mostazal, Chile (Globalmet 2021). Temperature and rain data from the Parlier, CA trial site is provided by the Kearney Agricultural Research and Extension Center (University of California 2021). Rainfall data and temperature data from Trial 4 is provided by the NOAA/NCDC National Centers for Environmental Information, Asheville North Carolina from their website at ncdc.noaa.gov, from the weather stations GHCND:US10hami004 (Menne et al. 2012a) and GHCND:USW00014935 (Menne et al. 2012b), respectively. Rainfall and temperature data from the Sorghum Trial is provided by the Front Compound Weather Data Station at the Pee Dee Research and Education Center (Clemson 2021).

| Trial 1 | | | Trial 2 | | | Trial 3 | | | Trial 4 | | Sorghum Trial | |
| --- | --- | --- | --- | --- | --- | --- | --- | --- | --- | --- | --- | --- |
|  | Genotype | N |  | Genotype | N |  | Genotype | N | Genotype | N | Genotype | N |
| Drought | H001 | 39 | Well-watered | H004 | 46 | Double Drought | H015 | 49 | H015 | 41 | S1 | 83 |
|  | H002 | 44 |  | H005 | 43 |  | H016 | 48 | I007 | 39 | S2 | 37 |
|  | H003 | 44 |  | H006 | 45 |  | H005 | 45 | I002 | 36 | S3 | 38 |
|  | H014 | 44 |  | H007 | 41 |  | H007 | 48 | I003 | 39 | S4 | 34 |
|  | I001 | 44 |  | H008 | 21 |  | H008 | 48 | H016 | 41 | S5 | 38 |
|  | I002 | 44 |  | H009 | 32 |  | H010 | 49 | H019 | 38 | S6 | 15 |
|  | I003 | 44 |  | H010 | 45 |  | H014 | 50 | H020 | 38 | S7 | 17 |
|  | I004 | 44 |  | H011 | 49 |  | H013 | 49 | H021 | 39 | S8 | 18 |
|  | I005 | 44 |  | H012 | 45 |  | H017 | 50 | H022 | 37 | S9 | 16 |
|  | I006 | 44 |  | H013 | 22 |  | H018 | 47 | H023 | 31 | S10 | 53 |
| Well-watered | H001 | 45 |  | H014 | 32 | Single Drought | H015 | 46 | H024 | 38 | S11 | 53 |
|  | H002 | 45 |  | H002 | 45 |  | H016 | 49 | H025 | 39 | C | 7 |
|  | H003 | 41 | Water-limited | H004 | 20 |  | H005 | 49 | H026 | 39 |  |  |
|  | H014 | 45 |  | H005 | 27 |  | H007 | 50 | H027 | 39 |  |  |
|  | I001 | 46 |  | H006 | 16 |  | H008 | 50 | I001 | 40 |  |  |
|  | I002 | 44 |  | H007 | 23 |  | H010 | 49 | H028 | 38 |  |  |
|  | I003 | 45 |  | H008 | 21 |  | H014 | 48 | H001 | 42 |  |  |
|  | I004 | 46 |  | H009 | 22 |  | H013 | 48 | H004 | 40 |  |  |
|  | I005 | 45 |  | H010 | 20 |  | H017 | 49 | H005 | 40 |  |  |
|  | I006 | 44 |  | H011 | 18 |  | H018 | 50 | H029 | 39 |  |  |
|  |  |  |  | H012 | 25 | Well-watered | H015 | 50 | H006 | 39 |  |  |
|  |  |  |  | H013 | 22 |  | H016 | 49 | H007 | 41 |  |  |
|  |  |  |  | H014 | 20 |  | H005 | 49 | H008 | 40 |  |  |
|  |  |  |  | H002 | 21 |  | H007 | 46 | H030 | 40 |  |  |
|  |  |  |  |  |  |  | H008 | 48 | H009 | 41 |  |  |
|  |  |  |  |  |  |  | H010 | 47 | H011 | 42 |  |  |
|  |  |  |  |  |  |  | H014 | 50 | H031 | 41 |  |  |
|  |  |  |  |  |  |  | H013 | 49 | H014 | 39 |  |  |
|  |  |  |  |  |  |  | H017 | 50 | H032 | 40 |  |  |
|  |  |  |  |  |  |  | H018 | 48 | H033 | 39 |  |  |
|  |  |  |  |  |  |  |  |  | H013 | 35 |  |  |
|  |  |  |  |  |  |  |  |  | H017 | 39 |  |  |
|  |  |  |  |  |  |  |  |  | H018 | 39 |  |  |
|  |  |  |  |  |  |  |  |  | H034 | 34 |  |  |
|  |  |  |  |  |  |  |  |  | H003 | 42 |  |  |
|  |  |  |  |  |  |  |  |  | I005 | 38 |  |  |
|  |  |  |  |  |  |  |  |  | I004 | 41 |  |  |
|  |  |  |  |  |  |  |  |  | H002 | 39 |  |  |

Supplemental Table 3. Number of RootTrackers (N) by genotype and treatment for each trial. For Trials 1 and 2, only Version 2 RootTrackers are included in the count. RootTrackers in all other trials are Version 2 only.
